# Integration of Patient-Reported Outcome Data Collected Via Web Applications and Mobile Apps Into a Nation-Wide COVID-19 Research Platform Using Fast Healthcare Interoperability Resources: Development Study

**DOI:** 10.2196/47846

**Published:** 2024-02-27

**Authors:** Johannes Benedict Oehm, Sarah Luise Riepenhausen, Michael Storck, Martin Dugas, Rüdiger Pryss, Julian Varghese

**Affiliations:** 1 Institute of Medical Informatics University of Münster Münster Germany; 2 Institute of Medical Informatics Heidelberg University Hospital Heidelberg Germany; 3 Institute of Clinical Epidemiology and Biometry University of Würzburg Würzburg Germany

**Keywords:** Fast Healthcare Interoperability Resources, FHIR, FHIR Questionnaire, patient-reported outcome, mobile health, mHealth, research compatibility, interoperability, Germany, harmonized data collection, findable, accessible, interoperable, and reusable, FAIR data, mobile phone

## Abstract

**Background:**

The Network University Medicine projects are an important part of the German COVID-19 research infrastructure. They comprise 2 subprojects: COVID-19 Data Exchange (CODEX) and Coordination on Mobile Pandemic Apps Best Practice and Solution Sharing (COMPASS). CODEX provides a centralized and secure data storage platform for research data, whereas in COMPASS, expert panels were gathered to develop a reference app framework for capturing patient-reported outcomes (PROs) that can be used by any researcher.

**Objective:**

Our study aims to integrate the data collected with the COMPASS reference app framework into the central CODEX platform, so that they can be used by secondary researchers. Although both projects used the Fast Healthcare Interoperability Resources (FHIR) standard, it was not used in a way that data could be shared directly. Given the short time frame and the parallel developments within the CODEX platform, a pragmatic and robust solution for an interface component was required.

**Methods:**

We have developed a means to facilitate and promote the use of the German Corona Consensus (GECCO) data set, a core data set for COVID-19 research in Germany. In this way, we ensured semantic interoperability for the app-collected PRO data with the COMPASS app. We also developed an interface component to sustain syntactic interoperability.

**Results:**

The use of different FHIR types by the COMPASS reference app framework (the general-purpose FHIR Questionnaire) and the CODEX platform (eg, Patient, Condition, and Observation) was found to be the most significant obstacle. Therefore, we developed an interface component that realigns the Questionnaire items with the corresponding items in the GECCO data set and provides the correct resources for the CODEX platform. We extended the existing COMPASS questionnaire editor with an import function for GECCO items, which also tags them for the interface component. This ensures syntactic interoperability and eases the reuse of the GECCO data set for researchers.

**Conclusions:**

This paper shows how PRO data, which are collected across various studies conducted by different researchers, can be captured in a research-compatible way. This means that the data can be shared with a central research infrastructure and be reused by other researchers to gain more insights about COVID-19 and its sequelae.

## Introduction

### Background

Owing to the high number of individuals infected with SARS-CoV-2, lasting symptoms and sequelae are becoming increasingly common in the global population. Therefore, research on this topic is important. The clinical picture of these patients is usually referred to as post–COVID-19 condition. Regular follow-ups and continuous monitoring of patients with post–COVID-19 condition are crucial to collect data for research about this new disease.

As treatment for patients with post–COVID-19 condition is typically done in the home setting, the use of electronic patient-reported outcomes (ePROs) is the preferred means of collecting data about symptoms, well-being, and overall health, which is key for follow-up and gathering more information.

### Objective

As aftercare and data gathering is time consuming and associated with high costs, sharing and reusing the data for multiple research projects is very beneficial. Therefore, it is necessary to provide a means to collect interoperable data and to create an infrastructure to exchange data.

During the peak of the pandemic, the Network University Medicine (NUM) was founded in Germany to create a central research infrastructure to collect and share medical data on COVID-19. As part of a small subproject, an app framework for ePRO collection was developed by a group of research experts and technical experts who had previous experience in conducting studies with patient-reported outcomes (PROs) and electronic data capture systems.

To reinforce the benefits of the research infrastructure provided by NUM, the objective of this study was to integrate the data collected by the ePRO apps into the central data repository. Therefore, an interface component (compass-interface-codex) was developed.

## Methods

### Prerequisites

When the first lockdown was imposed at the start of the COVID-19 pandemic, smartphones became an important tool for data collection. Researchers urgently needed data to better assess the typical natural history of the new disease owing to its threat to public health and the psychological and social impact of the lockdowns and social distancing measures.

At that time, many apps were developed at very short notice. They were often developed as isolated solutions for comparatively similar or even the same research problems, but the data were neither standardized nor interoperable, which limited their scientific use [1]. Hence, the Coordination on Mobile Pandemic Apps Best Practice and Solution Sharing (COMPASS) project was initiated by the German Federal Ministry of Education and Research early during the pandemic. In this project, interdisciplinary teams of experts came together and collected the best practices for the development and deployment of pandemic apps in terms of technical, ethical, and regulatory requirements.

**Figure 1:**
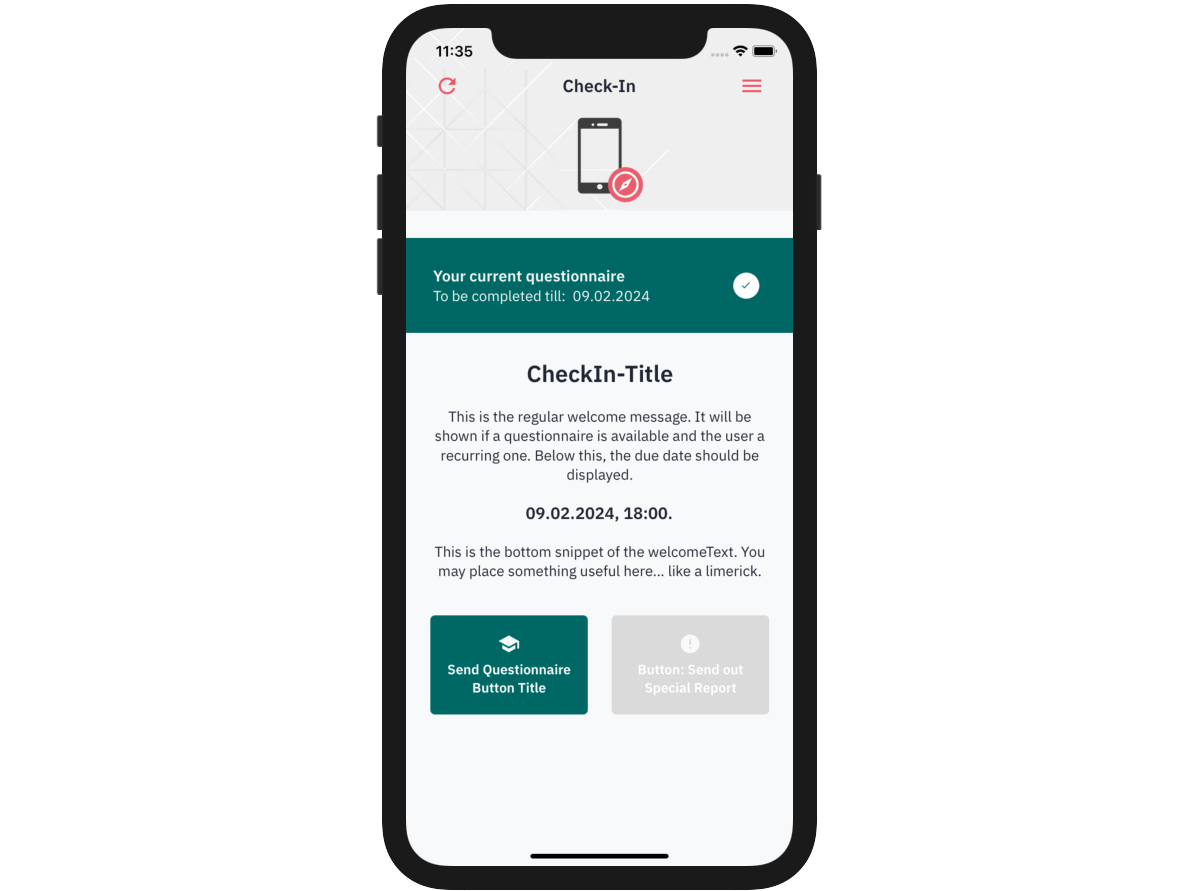
Main page of the Coordination on Mobile Pandemic Apps Best Practice and Solution Sharing reference app with default styling.

As part of the COMPASS project, a reference app framework for ePROs was developed, consisting of a native app and a web application. A screenshot of the noncustomized COMPASS native app with the default style is shown in Figure 1. A questionnaire editor was also developed to configure the apps. The app framework is available as open-source software on GitHub [2] and can be used and customized by anyone free of charge.

COMPASS is part of the NUM projects, which was established specifically for COVID-19 research through special funding from the German Federal Ministry of Education and Research as part of the crisis management for COVID-19 research. All 36 university hospitals in Germany are involved. A total of 13 projects were funded, including COMPASS and COVID-19 Data Exchange (CODEX) [3].

The German Corona Consensus (GECCO) data set [4] is a COVID-19–related core data set. It was defined at the start of the NUM projects to enable harmonized cross-site and cross-project data capture. The GECCO data set is defined in *Advanced Requirement Tooling using Data Elements, Codes, Object Identifiers and Rules*, and a set of Fast Healthcare Interoperability Resources (FHIR) profiles covering the Patient, Consent, Observation, Condition, Procedure, and MedicationStatement resource types. The elements are coded using *International Statistical Classification of Diseases and Related Health Problems-10th edition–German Modification* (*ICD-10–GM*), Logical Observation Identifiers Names and Codes (LOINC), Unified Code for Units of Measure, Anatomical Therapeutic Chemical (ATC), and Systematized Nomenclature of Medicine Clinical Terms (SNOMED CT).

Another important infrastructure project by NUM is the CODEX platform [5]. A secure and extensible platform was developed to collect GECCO data from the data integration centers of the German Medical Informatics Initiative [6] in a central location. This prevents the creation of data silos at local sites. There is a portal through which researchers can request access to the data. The CODEX platform is hosted in a secure environment at the society for scientific data processing (Gesellschaft für wissenschaftliche Datenverarbeitung mbH Göttingen) [7].

### Requirement Analysis

Before the technical requirement analysis, a group of experts within NUM-COMPASS gathered the requirements for research professionals.

**Figure 2:**
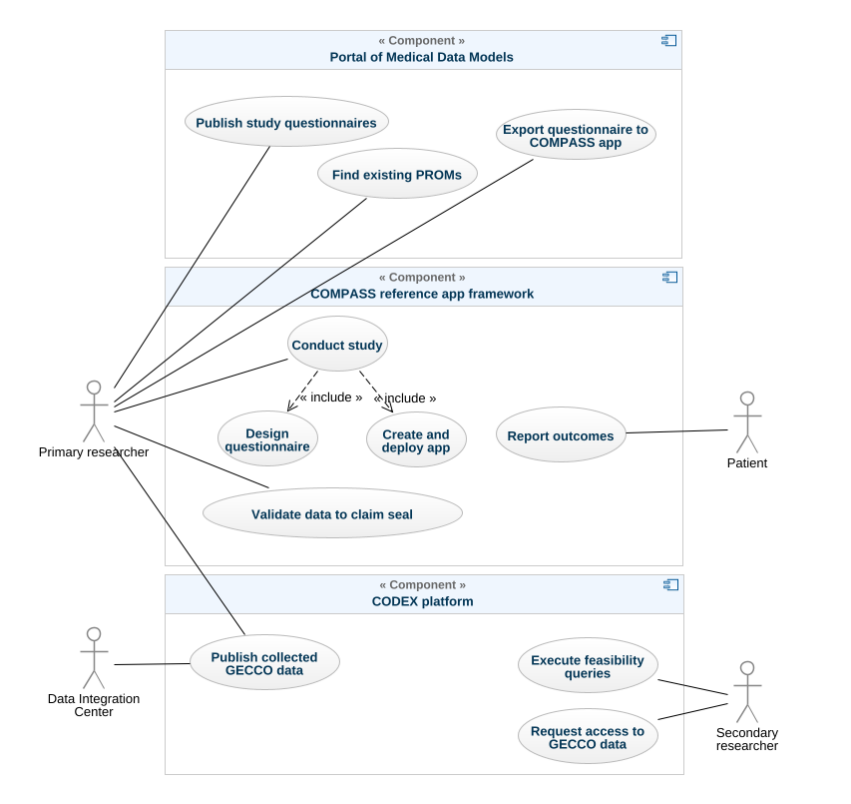
Use case diagram with the involved actors and components. CODEX: COVID-19 Data Exchange; COMPASS: Coordination on Mobile Pandemic Apps Best Practice and Solution Sharing; GECCO: German Corona Consensus; PROM: patient-reported outcome measure.

An overview of the use cases, including the components and actors involved, is illustrated in Figure 2. Researchers as primary end users want to use the COMPASS app framework to conduct a study by designing questionnaires and deploying the app to capture data provided by the patients for their research. The captured data can then be published on the CODEX platform, so that others can perform secondary research. Therefore, data must be transformed between the data formats used by COMPASS and CODEX.

At this point, apart from the technical transformation, which is usually cumbersome but feasible after data collection, the incompatibility of the data elements collected across various studies was considered a major problem. Therefore, the use of core data sets was emphasized from the beginning. As the use of the GECCO data set was already defined for all NUM projects, it was a logical decision to promote its use. This means that if a new study commences, the data items to be collected should use the GECCO data set as a starting point.

**Figure 3:**
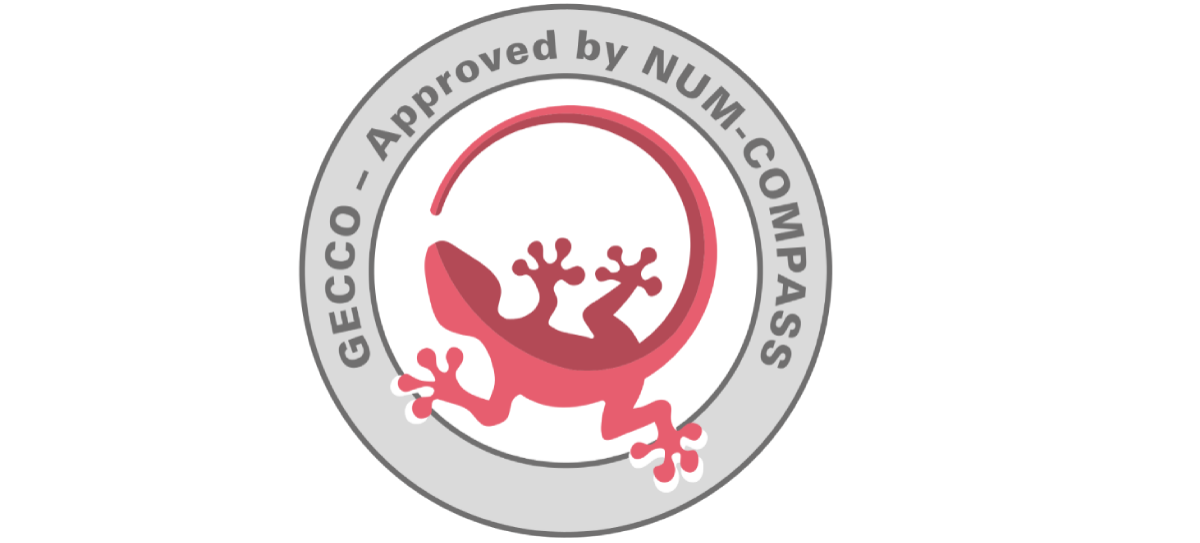
German Corona Consensus (GECCO)–approved seal that can be issued to studies conducted using the GECCO data set.

Lack of awareness, lack of time, and lack of direct benefit were considered the main reasons why researchers ignore the need to capture data in a research-compatible way. To mitigate these effects, a First Contact Package [8] for COMPASS users was developed to explain good practices to researchers and recommend using the GECCO data set. To incentivize the use of the GECCO data set, a GECCO – Approved by NUM-COMPASS seal (Figure 3) was developed. To award this seal, Data4Life was tasked to develop a validation service called GECCO Conformance Checker [9].

To encourage the reuse of further study-specific data items beyond the GECCO data set, the Portal of Medical Data Models (MDM-Portal) [10] was recommended, and resources should be provided to facilitate integration. The MDM-Portal is a research infrastructure that provides public access to 25,157 data models (as of July 2023).

For cases in which the study has already started with a different data set, a “Late Mapping Scheme” to the GECCO data set has been created, but the emphasis is on using the GECCO data set from the beginning.

### Technical Evaluation of Existing Components

The first step in developing the interface component was to examine the reference app framework.

The reference app framework consists of source code templates for building a native app and a web application. The native app template is based on the Gutenberg COVID-19 study [11] app and was developed by IBM Corp. The web application is based on CovApp developed by Data4Life. The questionnaire editor was developed from scratch by Healex Systems.

To start a new study with the app framework, a developer can fork the existing source code from GitHub and exchange icons and textual description templates. Extensive documentation and video tutorials [12] are available for reference. For customization, new features can be added to the implementation, which can then be published in the Apple App Store and Google Play Store.

To participate in a study, patients need to install the custom-built app from their smartphone’s app store. To authorize the installed app for participation in the study, the patients have to scan a personalized QR code, which transmits the SubjectID to the device. Then, the app retrieves the questions from FHIR Questionnaire resources from the back end. These questions are then displayed to the user on a regular schedule.

After the user responses are collected, the app generates an FHIR QuestionnaireResponse resource that is sent as an asymmetrically encrypted binary large object (BLOB) and stored in the back end. Only researchers who have access to the private key can decrypt the BLOB with a Python downloader script, whereas the private key is not accessible in the back end.

Public cloud services can be used, as the responses remain encrypted in the app back end. This eliminates the need for the time-consuming configuration of firewall exceptions in hospital networks, increases the availability of the app back end, and saves hosting costs. Another major benefit is that it enables the use of cloud-based application programming interfaces for sending push notifications. This way, users can be automatically notified when new questionnaires need to be completed, which likely results in an increase in response rates [13].

The CODEX platform uses an EHRbase [14] instance as an openEHR-based central storage system. The transmission is done via an FHIR interface, but no Questionnaire resources are used. Instead, the platform requires FHIR resources, which are compliant to the GECCO profiles to receive the data. Using the FHIR Bridge [15], the GECCO profiles are mapped to the corresponding openEHR templates specifically created for the GECCO data set and stored in an EHRbase instance. This way, the data stored in CODEX are compatible not only with FHIR but also with openEHR.

### Technical Requirement Analysis

Both the app and CODEX are using FHIR-based [16] interfaces for data exchange. Health Level–7 FHIR is an emerging standard in the field of health research [17]. FHIR is built upon XML, JSON, and Representational State Transfer technologies, which are comparatively lightweight and therefore well suited for mobile apps. The standard defines a collection of >140 resource types that define clinical concepts. These resource types can be adapted and used in a variety of use cases. The use of these resources to solve a specific interoperability problem is described in the implementation guides. In these implementation guides, FHIR resource types are usually constrained using the profiling mechanism.

Interoperability may require the conversion of data within the FHIR standard. In our case, there will be a variety of FHIR Questionnaires used in the apps and a different set of resource types constrained by the GECCO profiles used by the CODEX platform to exchange data. The gap in between was identified as the biggest obstacle.

A FHIR Questionnaire resource stores only structural information. It consists of items that can be nested hierarchically. These items consist primarily of the text displayed to the user, the answer’s data type, visibility conditions (enableWhen), the “required” attribute, and the linkId. The linkId is used for linkage between the answer in the QuestionnaireResponse and the corresponding question item in the Questionnaire.

The resource types constrained by the GECCO profiles (Patient, Observation, Condition, etc) are much stricter: They usually consist of a central code element linked to an external medical terminology such as LOINC, SNOMED CT, ATC, or *ICD-10–GM*. Therefore, they enforce a much higher degree of semantic interoperability of data. This is in contrast to Questionnaire and QuestionnaireResponse resources, which, on the basic level, only enforce syntactic interoperability.

The COMPASS apps use FHIR Questionnaires to keep the questions from various studies reusable. However, the CODEX platform cannot process FHIR Questionnaires of unknown origin. It is possible to store specific questionnaires in the FHIR Bridge and create a manual mapping to an openEHR template that has to be created specifically for the Questionnaire.

Zhu et al [18] used this approach to integrate the COVID-19 patient assessment questionnaire with the native app, which had already been integrated in the CODEX platform for data collection with CovApp [19]. For new studies with different questionnaires, this approach to integrate requires a lot of work on the CODEX platform for the connection of each new app, and it will not work if a study introduces new questionnaires or if existing questionnaires are changed at short notice.

In conclusion, to solve the interoperability problem, it is necessary to transform the items from the QuestionnaireResponses to their corresponding resources in the GECCO implementation guide, which is applicable for new studies conducted with the COMPASS app framework.

### Point of Transformation

Before the implementation of an interface component could begin, an architectural decision had to be made about the location of the conversion from FHIR QuestionnaireResponse to the GECCO profiles.

The various options were identified and evaluated in an expert-led roundtable with the back end, native app, and web application developers.

### Assessment of Existing Mapping Methods

To address the question of how items from QuestionnaireResponses can be mapped to the corresponding FHIR resource types, a literature search was performed by using the keywords “FHIR” and “Questionnaire” in the PubMed and MEDLINE databases. Furthermore, an in-depth examination of the FHIR standard was performed.

### Evaluation

To verify the correctness of the mapped resources, the GECCO Conformance Checker developed by Data4Life was used. A test data set containing 10 patients with 20 QuestionnaireResponses was submitted to the central platform to verify the overall pipeline.

### Ethical Considerations

All data used during the development and implementation process were synthetic. No data of real persons were used; therefore, ethics approval was not required.

## Results

### Point of Transformation

In the expert round, the following options were identified and their respective advantages and disadvantages were discussed:

1. On the smartphone: This would require 2 implementations for both the native app and the web application, thereby increasing costs. In addition, this solution would increase the download size of the apps but without adding any direct value for the patient. Owing to this fact and the risk of additional problems that could arise from outdated app versions transmitting outdated mappings, this option was discarded.
2. Inside the back end of the COMPASS project: Both the native app and the web application use the same back end for data submission. Thus, the back end would also be conceivable as the location of the transformation. However, this would require decryption of the QuestionnaireResponse BLOBs within untrusted cloud servers. This option would offset the privacy gains of the encrypted architecture and therefore had to be rejected.
3. What remained was the development of a separately running custom component that would run within the protected environment of the CODEX platform. Therefore, this component polls the common app back end periodically, downloads the encrypted BLOBs, decrypts them, and stores the transformed data in the internal data storage layer.

**Figure 4:**
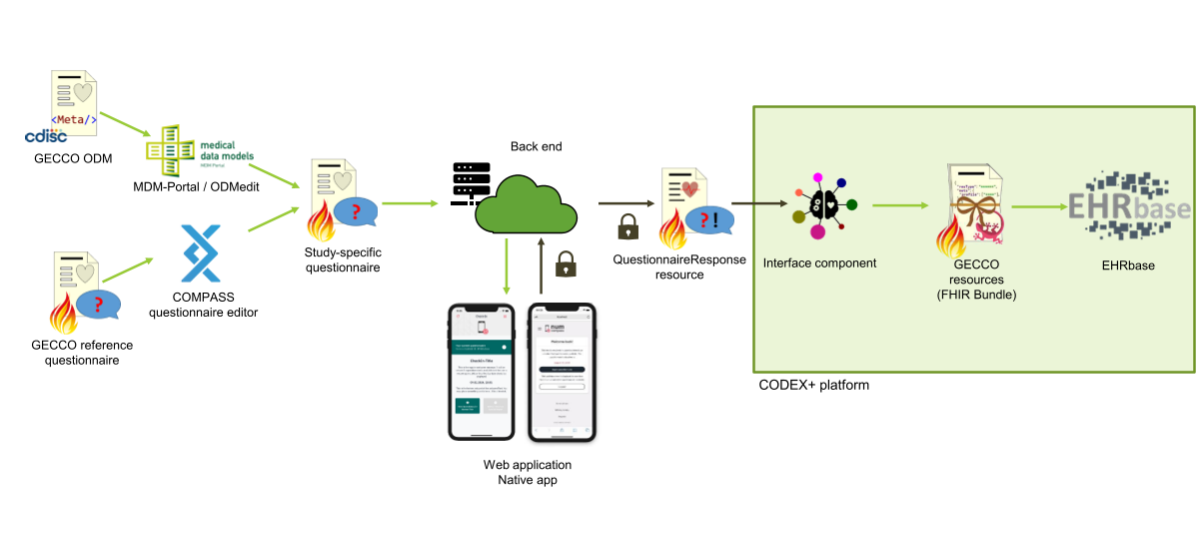
Overview of the overall architecture and the involved resource types and software components. The arrows indicate the data flow. Green arrows indicate that the data are only transport encrypted, whereas the brown arrows indicate the end-to-end encryption of the QuestionnaireResponse resource throughout the cloud-hosted back end. CODEX: COVID-19 Data Exchange; FHIR: Fast Healthcare Interoperability Resources; GECCO ODM: German Corona Consensus Operational Data Model; MDM: Medical Data Model.

The final architecture and the flow of data are shown in Figure 4.

### Assessment of Existing Mapping Methods

The literature search, as of September 1, 2021, returned the following results:

1. MEDLINE: 27 hits in the search for FHIR Questionnaire; none of them was relevant
2. Web of Science: 11 hits; none of them was relevant

In a publication, a mapping of a logical model that resulted from natural language processing parsing to other FHIR resource types was apparently performed [20].

The FHIR standard itself does not provide any suggestions about how to extract FHIR resources, but the Structured Data Capture Implementation Guide contains 3 different proposals—observation based, definition based, and FHIR Mapping Language [21]. All of them had different shortcomings for our use case, which are outlined in Multimedia Appendix 1, and an adaptation could not be done in a timely manner. Therefore, it was decided to perform the mapping with the Kotlin programming language.

### Mapping Concept and Implementation

In the FHIR context, so-called logical models are commonly used to define medical data models by domain experts with minimal knowledge about FHIR resources. These logical models are later “translated” into FHIR profiles [22]. As GECCO’s logical model is structurally more similar to a questionnaire than the GECCO FHIR profiles, we used it as the starting point for a project-specific logical model [23] and used the questions of the REDCap (Research Electronic Data Capture; Vanderbilt University) forms provided by Erbelding et al [24], which were developed for capturing GECCO data sets in hospitals, to develop the logical model further, so that it can be translated into a questionnaire.

Our logical model contains all the medical concepts and data elements included in the GECCO data set. Each data element is annotated with the corresponding data type and a textual question. The logical model was created with a well-defined mapping to the corresponding GECCO FHIR profiles in mind. An excerpt of the logical model is shown in Textbox 1.

**Excerpt of the structure of the logical model of the German Corona Consensus (GECCO) data set. Element identifier with associated questions are in parenthesis as well as the possible answer options.**

- anamnesis: („Anamnesis/risk factors“)
  hasChronicLungDisease („Does the patient suffer from a chronic lung disease?“): yes/no/unknown
  chronicLungDisease: („Which chronic lung disease does the patient suffer from?“, enableWhen anamnesis.hasChronicLungDisease = yes)
    asthma („Asthma“): yes/no/unknown
    copd („COPD“): yes/no/unknown
    (...)
  (...)
- demographics („Demographics“)
  biologicalSex („Biological sex“): male/female/unknown/x/d
  (...)
- epidemiologicalFactors („Epidemiological factors“)
  (...)
- complications („Complications“)
  (...)
- (...)

The implementation was performed using the Kotlin programming language and the *HL7 Application Programming Interface* FHIR library [25]. The decryption of the encrypted QuestionnaireResponse BLOBs was implemented using the bouncycastle library. A Helm chart, which integrates into the CODEX platform infrastructure, was created for deployment.

The logical model is stored as a set of Kotlin classes. The elements are annotated with textual questions and LOINC or *ICD-10–GM codes*, if applicable. A class structure rather than JSON-based data formats to define the logical model, for example, was chosen because several common patterns could be found in the GECCO data set, which required >1 Questionnaire item per profile. For these common patterns, custom data types such as *YesNoUnknownWithDateTime* (vaccinations) and *YesNoUnknownWithSymptomSeverity* (symptoms) were defined, which have a specific mapping and a well-defined enableWhen behavior and can be used at multiple places. For example, it is pointless to ask the patient about the severity of their cough if they have previously answered that they do not have a cough.

Another benefit of using a class structure is the resulting type safety inside the transformation logic. If the logical model is updated, the affected profiles can easily be found in the process.

To improve the usability of the Questionnaire, in the anamnesis part, meta-elements were defined, which do not directly correspond to profiles of the GECCO data set. Those meta-elements ask the user for a specific category of preexisting diseases. If the user answers “No” or “Unknown” for this disease category, the apps will not show the list of all diseases in the data set using the enableWhen mechanism and the Condition resources will be set to “No” or “Unknown”, respectively.

By means of reflection, the GECCO reference questionnaire is generated from the logical model. This questionnaire can be used by researchers as a starting point for editing a custom questionnaire. It is stored as a reference questionnaire in the questionnaire editor to be able to import individual items from the GECCO data set.

Each item in the generated reference questionnaire has an extension with a reference to the path in the logical model. This path is transferred into the QuestionnaireResponse resource by the COMPASS apps. For other apps, the interface component is also able to transfer the extensions into the QuestionnaireResponse resource if the Questionnaire resource is known. This way, individual items of the reference questionnaire can be uniquely identified via the path in the logical model. The mapping is then executed based on this assignment. The generated GECCO profile instances are exported in the form of an FHIR bundle.

In total, the GECCO data set could be broken into 45 template functions for the different profiles and resources, and >430 data elements coded with SNOMED CT, LOINC, ICD-10-GM, or ATC were created. The template functions are available as part of the internal library “gecco-easy”, which can be used for manual transformation of other Questionnaire items.

The reference to the subject of the created resources is automatically set by the SubjectID used in the app, and the recording time of the resources is automatically set to the time the QuestionnaireResponse was submitted (refer to Table 1 for specifically set attributes).

**Table 1:** Mapping of the contextual information of the QuestionnaireResponse to the corresponding attributes of the specific resource types.

FHIR^a^ resource type	Subject-specifying attribute	Recorded, time-specifying attribute
Patient	N/A^b^	N/A
Consent	Consent.patient	N/A
Observation	Observation.subject	Observation.effectiveDateTime
Condition	Condition.subject	Condition.recordedDate
Procedure	Procedure.subject	Procedure.performedDateTime^c^
MedicationStatement	MedicationStatement.subject	MedicationStatement.effectiveDateTime
Immunization	Immunization.patient	Immunization.occurenceDateTime^c^

^a^FHIR: Fast Healthcare Interoperability Resources.

^b^N/A: not applicable.

^c^For domain-specific reasons, these attributes are not inferred by the context.

A problem we encountered with FHIR Questionnaires was the lack of support for multiple Codings per answerOption in the Questionnaire. The Condition.code, Observation.code, and Procedure.code elements are of type CodeableConcept, which means that multiple Codings can be used to describe a fact. In most cases, 1 Coding is sufficient for a distinct assignment to 1 answer option in the logical model. From that point, the interface component can emit all the required Codings for the target resource profile. However, 2 custom CodeSystems had to be created (Covid19-Vaccine and VentilationTypes).

The implemented solution was documented as part of the COMPASS implementation guide [26]. This way, other apps can use the extensions and thus the interface component for their needs.

### Improvements to the Questionnaire Editor

To tackle the issue of lack of time that most researchers face during the Questionnaire development phase, an import function for GECCO items was added to the questionnaire editor. The import button is prominently placed among the other buttons to add new items. This way, it is hard to miss, and importing the existing GECCO items or item sets is more convenient than designing custom ones; however, it is still possible.

When a question or a set of questions is imported from the GECCO data set, it is highlighted accordingly in the user interface. Some functions such as adding or removing new answer options are disabled to avoid problems with different scales and the grade for symptoms.

**Figure 5:**
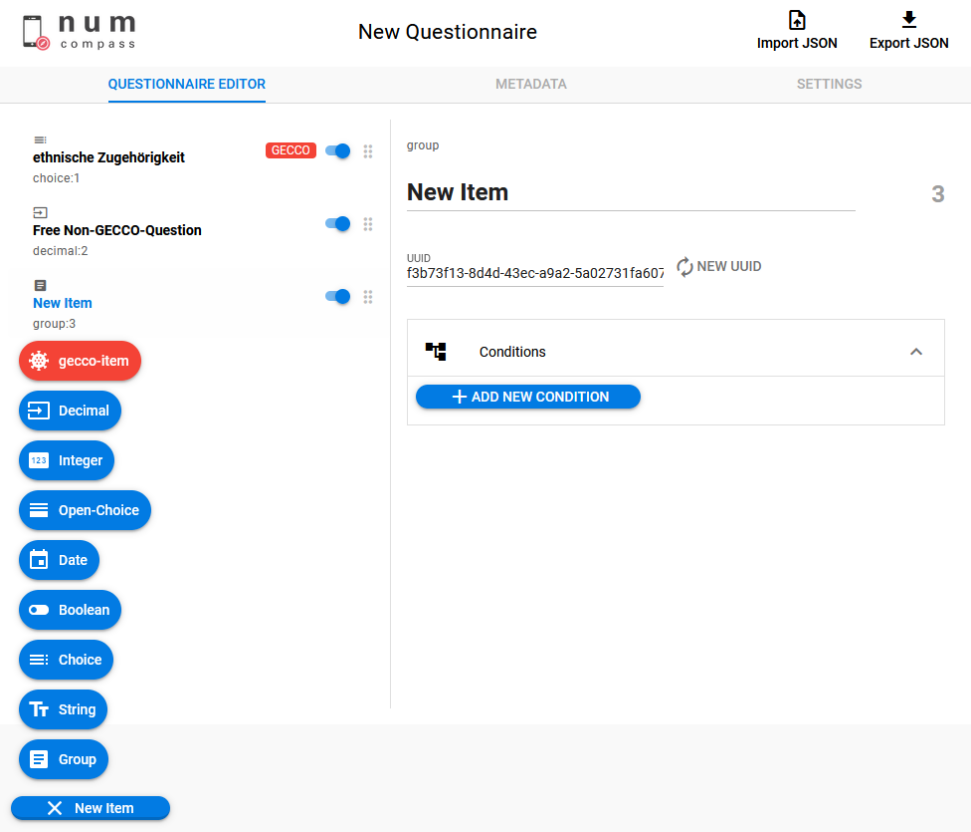
Screenshot of the Coordination on Mobile Pandemic Apps Best Practice and Solution Sharing questionnaire editor with German Corona Consensus (GECCO) and non-GECCO items inside a Fast Healthcare Interoperability Resources Questionnaire resource. On the left, the tree of questionnaire items is shown; on the right, the details of the selected item can be configured. The GECCO items are highlighted in the tree. The menu to add new items at the bottom left is expanded, revealing the option to add GECCO items along with the standard item data types.

A screenshot of the updated editor is shown in Figure 5.

### Integration With the MDM-Portal

To include additional PRO measures (PROMs), an integration with the MDM-Portal [27], which internally uses Clinical Data Interchange Standards Consortium Operational Data Model (ODM) files, was implemented.

To ensure interoperability with the existing apps, interface component, and GECCO FHIR profiles, a new variant of the existing ODM to FHIR Questionnaire converter [28] called ODM2COMPASS was created. For this purpose, the items within the GECCO form were annotated with their respective path in the logical model using ODM’s Alias-Tag. The ODM2COMPASS converter uses this annotation to create the CompassGeccoItem extension in the resulting Questionnaire, which is needed by the interface component to map the response to the corresponding GECCO profiles.

Further adaptations to the existing converter include a conversion of MDM-Portal’s adapted ODM-ConditionDef syntax to enableWhen element of FHIR Questionnaires. As FHIR Questionnaires only support 1 Coding per answerOption but ODM allows multiple aliases per CodeListItem, the reference Questionnaire had to be included. When there are multiple Codings or Alias-Tags available, the CodeSystem or Context used in the reference Questionnaire will be used in the export process by the converter. This way, the interface component can recognize not only the questions but also the answers. For provenance, the Questionnaire.url element is set to the MDM-Portal’s URL of the form.

Researchers can create custom questionnaires by creating a new data model and using the built-in ODMedit tool [29], which also allows for carrying over parts of other forms inside the MDM-Portal. As the Alias-Tags are also adopted, when the resulting form is exported to the reference app framework, the interface component is still able to recognize elements from the original GECCO form and map them to the corresponding profiles.

This way, researchers can easily combine the GECCO data set with PROMs that contain items that are not available in GECCO such as overall well-being (eg, World Health Organization-5 Well-Being Index) or mental health (eg, Generalized Anxiety Disorder 7). Furthermore, researchers can publish their newly created questionnaires directly in the portal, increasing the level of adherence to the FAIR (findability, accessibility, interoperability, and reuse) criteria in the process.

### Validation of the Created Resources

A generator script for QuestionnaireResponses was developed, which produces QuestionnaireResponses with random answers based on an input Questionnaire resource. These generated QuestionnaireResponses were then transformed into bundles of GECCO profiles by the interface component. This way, all possible answer options could be tested.

**Figure 6:**
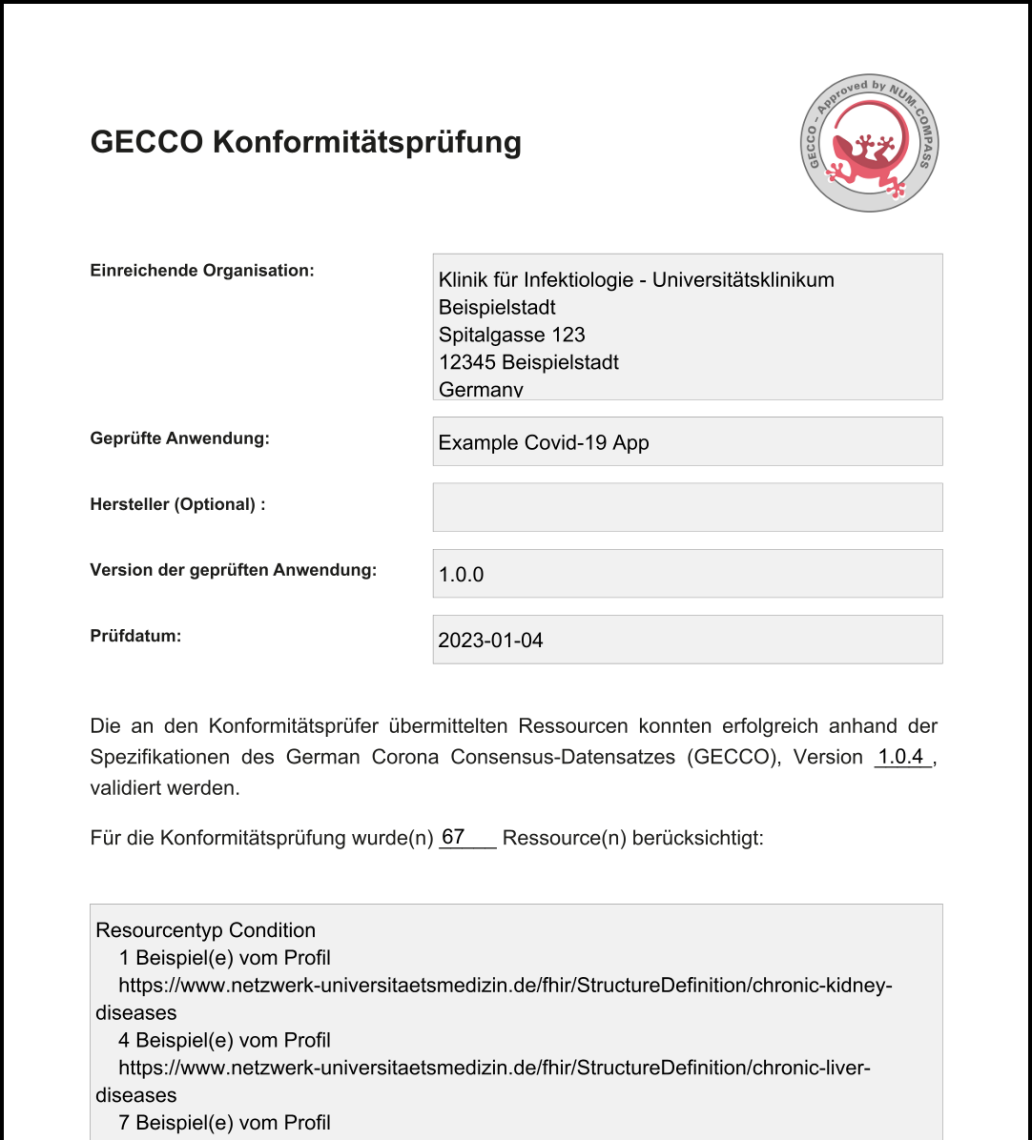
Screenshot of the result of the German Corona Consensus conformance check and comparison of the existing methods for Questionnaire extraction.

As part of the project, Data4Life was commissioned to develop the GECCO conformance checker. This component validates the entered resources for conformance with the FHIR profiles and creates a PDF file with the GECCO – Approved by NUM-COMPASS seal as an incentive for researchers [9]. A screenshot of such a validation result document can be seen in Figure 6. This conformance checker was used to verify whether, for each GECCO item, a resource was generated and all profiles were created correctly.

In addition, further validation was conducted using EHRbase and FHIR Bridge. Both components represent the storage layer of the CODEX platform. The FHIR Bridge provides an FHIR Representational State Transfer application programming interface, which accepts and transforms the GECCO profiles to their corresponding openEHR templates. The resulting openEHR composition is then stored in the EHRbase instance. To this end, a variety of manual transformation functions have been defined inside FHIR Bridge, which strictly assume that the submitted resources conform to the GECCO profiles.

The final integration test was performed using a synthetic test data set containing 10 patients with 20 QuestionnaireResponses covering various corner cases. The test data were successfully transferred to the CODEX platform, and the correct mapping was manually validated.

## Discussion

### Principal Findings

We successfully created a means to integrate GECCO data that are collected by the COMPASS app framework into the CODEX platform in a way that can be efficiently applied to a variety of questionnaires. The generalizability of our approach makes it superior to the existing approach used by Zhu et al [18], which requires using a fixed questionnaire with the apps and a questionnaire-specific implementation at the central platform, which severely limits its practical use.

Normally, this flexibility in the design of the questionnaires would require the manual creation of questionnaires by the researchers to obtain interoperability with the central platform. After the creation of questionnaires, it would be necessary to create a manual mapping script for each questionnaire to assign the questionnaire items to the GECCO items and create the corresponding FHIR resources. This requires clinicians to have deep technical knowledge about programming languages and FHIR profiles. In contrast, our solution requires clinicians to have much less technical knowledge. It includes an interface component, which is compatible with different questionnaires, as long as the researchers provide the correct annotations. To create questionnaires with the said annotations, we provide 2 different questionnaire editors.

The COMPASS questionnaire editor was tailored to the feature set of the native app and the web application. The capability of importing questions is limited to the GECCO data set. This can be seen as an advantage, as it increases the hurdles to use data sets competing with the GECCO data set that cannot be integrated into the CODEX platform.

However, because the GECCO extension modules are in an early stage, it is better to facilitate the reuse of existing PROMs for areas that are not yet covered by the GECCO data set. This is where ODMedit, with its existing integration to the MDM-Portal, can shine. The MDM-Portal provides a vast number of PROMs, which can be easily used to extend the GECCO data set. However, owing to the generic nature of ODMedit and the involved conversion step between ODM and FHIR, it does not have the capability to configure features that are specific to FHIR Questionnaires and the COMPASS apps.

A possible mitigation is using ODMedit to create the initial Questionnaire resource by importing items from the MDM-Portal and importing the Questionnaire into the COMPASS questionnaire editor to configure the exact behavior of the items in the app.

### Limitations

Notably, the integration work conducted still has limitations that must be considered. The most important limitation is that few studies have been conducted not only with the COMPASS framework itself but also with the integrated version. Even if PRO-based studies are becoming increasingly important, the acceptance of the users in the long term must always be considered. Currently, it is unclear if this acceptance will be provided. As long-term use has not yet occurred, relatively little data have been generated. Therefore, it is technically not yet possible to assess whether all aspects of data collection have been sufficiently taken into account. In addition, the health care sector is changing at a very fast pace, which may make aspects of integration obsolete. Nevertheless, from a standard perspective, the project presented is currently unique in our view and shows that open and standard-compliant development is possible in the fast-moving app sector.

On the technical side, in the current interface component implementation, the data elements of the logical model may only appear exactly once in the Questionnaire. The reason for this limitation is that only 1 instance of the logical model is created during the transformation process. Theoretically, it is possible to split the Questionnaire into small parts that are mapped independently.

Considering that the apps do not support repeatable elements and the current workflow of completing and submitting a questionnaire on the same day, this limitation is not relevant for practical use.

The interface component is constrained to the GECCO data set. This was done on purpose, in the context of the project and with the intent to enforce research compatibility. This means that other data captured by the studies cannot be transferred to the CODEX platform, limiting the use for answering questions beyond the GECCO data set. However, it is possible to adopt the basic principle of logical models to other research databases and their data models.

The development of the interface component was done with the focus on providing syntactic and semantic interoperability. Depending on the study data protection design and the consent given by the patient, additional pseudonymization steps involving an additional, trusted third party might be necessary before the interface component can be used.

### FHIR and FHIR Questionnaires

On the basis of our experience in the project, there was often the misconception, especially among clinicians, that if 2 systems “speak” FHIR, they are automatically compatible without considering the use of different implementation guides, resource types, or profiles to solve a specific problem.

We believe that through the use of 140 common resource types as building blocks, FHIR provides a solid foundation for interoperability in health care. However, FHIR is not a “panacea”, as there is the ongoing as there is the ongoing challenge of building bridges between different implementation guides, as seen in our case.

Given the increasing prevalence of the FHIR standard in both electronic data capture systems and other health information systems and the growing demand for interoperable solutions, closing the gap between questionnaires and other resource types is of great importance.

We believe that there are many use cases for mapping the data captured with questionnaires to other resource types for exchange with other existing systems.

It seems very promising to promote this by reusing the existing item blocks for new questionnaires that use some sort of annotation for mapping to other resource types. Templating mechanisms (importing item trees from other questionnaires) were nonexistent during the project’s requirement analysis phase but seem to be of great interest, as was recently shown by the specification of the $assemble operation by the FHIR Structured Data Capture working group [30].

### Outlook and Further Studies

The submission of GECCO FHIR resources is not limited to the CODEX platform. As GECCO profiles are also compliant with the German basic profiles, they can be integrated into a variety of FHIR-compatible software systems used in hospitals. For example, the responsible physician could monitor their patient’s well-being with familiar software tools.

We will monitor the development of the GECCO data set extension modules [31] and add them to the interface component once new FHIR profiles are released.

### Conclusions

We successfully created a concept to integrate PRO data into the German COVID-19 research infrastructure and implemented it.

Although educating and sensitizing researchers about syntactic and semantic interoperability during the design phase of a study already remains a top priority, we are convinced that the readiness for interoperable data collection can be increased as we provide researchers with a free, easy-to-use app framework that covers the semantic and technical aspects of integration. This will likely lead to the collection of more data in an interoperable way and contribute to secondary research.

Quickly gathering data about suddenly emerging infectious diseases such as monkey pox (“pandemic preparedness”) and overseeing the aftereffects of the COVID-19 pandemic such as post–COVID-19 condition are becoming high priority for public health. Therefore, future contributions to knowledge gain through PRO apps are likely to increase. National data registries, which are fed by multiple sources such as PROs that collect data in a unified format, can play an important role by improving health research and therefore will benefit patients in the long run.

## Appendix

Multimedia Appendix 1 Comparison of the existing methods for questionnaire extraction.

## Abbreviations

ATC: Anatomical Therapeutic Chemical
BLOB: binary large object
CODEX: COVID-19 Data Exchange
COMPASS: Coordination on Mobile Pandemic Apps Best Practice and Solution Sharing
ePRO: electronic patient-reported outcome
FAIR: findability, accessibility, interoperability, and reuse
FHIR: Fast Healthcare Interoperability Resources
GECCO: German Corona Consensus
*ICD-10–GM*: *International Statistical Classification of Diseases and Related Health Problems-10th edition–German Modification*
LOINC: Logical Observation Identifiers Names and Codes
MDM-Portal: Portal of Medical Data Models
NUM: Network University Medicine
ODM: Operational Data Model
PRO: patient-reported outcome
PROM: patient-reported outcome measure
REDCap: Research Electronic Data Capture
SNOMED CT: Systematized Nomenclature of Medicine Clinical Terms

## Data Availability

The source code of the interface component itself is available on GitHub [32]. The back end code of the Coordination on Mobile Pandemic Apps Best Practice and Solution Sharing app can be found in the same repository [33]. The Helm charts and the configuration of the COVID-19 Data Exchange platform are not open source but EHRbase [34] and FHIR Bridge [35] as the main components of the central platform can be used independently and are available in their corresponding GitHub repositories.
