# Supplementary material for: Integration of Patient-Reported Outcome Data Collected Via Web Applications and Mobile Apps Into a Nation-Wide COVID-19 Research Platform Using Fast Healthcare Interoperability Resources: Development Study
Source: J Med Internet Res. 2024 Feb 27;26:e47846. doi: 10.2196/47846 (PMC10933715; doi:10.2196/47846)
Supplement: Multimedia Appendix 1 [file jmir_v26i1e47846_app1.pdf]

Comparison of the existing methods for Questionnaire extraction

	Can create complex object types	Reusable blocks
Observation-based	(-)	(+)
Definition-based	(-)	(+)
StructureMap-based	(+)	(-)

- Observation-based: Here, single items are marked with the observationExtract extension. Then, in the extraction process, an Observation resource is created for each marked item. The Observation.code.coding will be set to the Questionnaire.item.code element, and the value of Observation.value[x] will set to the selected answer. All other Observation attributes like Observation.subject are inferred from the context of the questionnaire.

  In the GECCO data set, only 13 profiles are derived from Observation and only 10 of them could be extracted using Observation-based Extraction. The reasons were include CodeableConcepts that require multiple codings (PregnancyStatus) to describe a fact in the GECCO profiles, and the fact, that the common pattern of Yes/No/Unknown answer options (e.g. Known-Exposure) do not directly correspond to Observation.value[x], but require the emission of an extension in the target profile depending on the answer.
- Definition-based: With this approach, the itemExtractionContext-extension is added at the item group level to define the context for extracting a single resource from the subtree. Each item within this group then indicates the attribute of the target resource to which it should be mapped using the item.definition element. This approach can be used for any FHIR resource type and is not limited to the Observation.

  Although this approach is much more flexible, it still imposes several weaknesses: First, for each attribute in the target profile, which is fixed, a hidden elements must be created and set with an initial value. This inflates the Questionnaire size and worsens loading times and performance for the user. Hiding elements in FHIR Questionnaires requires an extension, so using the created Questionnaire with applications that do not support the extension would show a troublesome representation. Additionally, the primary researches direct analysis, which is usually performed directly using the QuestionnaireResponses would get much more complicated due to the large amount of unused fixed-value or calculated-value items.

  For profiles where the value of the user's response is not directly set to a

field in the GECCO resource (like with Yes/No/Unknown questions for example), it would be necessary to use calculated-expressions using FHIRPath based on the user's response to calculate these values. However, FHIRPath cannot be used to create complex data types such as extensions or CodeableConcepts, which would have to be written to the target profile depending on the user's response.

Another disadvantage is that the calculations for the mapping are already performed when the QuestionnaireResponse is created. This increases the development effort of the apps and the amount of data which has to be transmitted.

Even though this part of the implementation guide is the most promising, several custom extensions to FHIRPath would be needed. The short-term time horizon of the project prohibited such extensions. In addition, the integration with the MDM portal would not have been possible in this way, as it would require bloating not only the reference Questionnaire, but also the ODM file stored in the portal.

- StructureMap-based: FHIR specifies the lesser known StructureMap resource type to store the generic mapping between different hierarchical structures. A StructureMap resource can be created either directly using the respective JSON or XML representations, which would be very cumbersome, or by compiling a domain specific language called FHIR Mapping Language. The StructureMap resource can be executed using a mapping engine to do the transformation.

  The extension questionnaire-targetStructureMap, defined by the SDC implementation guide, could be used to link to a questionnaire to a corresponding StructureMap for data extraction. According to profile defined for the extension, the use of the StructureMap is only allowed on the level of the whole questionnaire, usage on items or item level is forbidden. Even though it is possible to create and import other StructureMaps for parts of the Questionnaires, this would still require always creating a custom StructureMap for that Questionnaire.

  Currently, only the HAPI FHIR library provides an open source implementation of a compiler for the FHIR Mapping Language and a Mapping Engine. To our experience, the degree of stability of this component seems to be rather low. At the same time, the FHIR mapping language seems to have a high degree of complexity compared to normal programming languages even for simple mapping tasks.
